# Dataset of gallic acid quantification and their antioxidant and anti-inflammatory activities of different solvent extractions from Kacip Fatimah (*Labisia pumila* Benth. & Hook. f.) leaves

**DOI:** 10.1016/j.dib.2023.109644

**Published:** 2023-10-05

**Authors:** Mohd Azrie Awang, Mohammad Amil Zulhilmi Benjamin, Adilah Anuar, Mohd Fakhrulddin Ismail, Shiamala Devi Ramaiya, Siti Nur Aisyah Mohd Hashim

**Affiliations:** a Faculty of Food Science and Nutrition, Universiti Malaysia Sabah, Jalan UMS, 88400 Kota Kinabalu, Sabah, Malaysia; b Innovative Food Processing and Ingredient Research Group, Faculty of Food Science and Nutrition, Universiti Malaysia Sabah, Jalan UMS, 88400 Kota Kinabalu, Sabah, Malaysia; c Borneo Research on Algesia, Inflammation and Neurodegeneration (BRAIN) Group, Faculty of Medicine and Health Sciences, Universiti Malaysia Sabah, Jalan UMS, 88400 Kota Kinabalu, Sabah, Malaysia; d Faculty of Chemical Engineering and Technology, Universiti Malaysia Perlis, Kampus UniCITI Alam, Sungai Chuchuh, 02100 Padang Besar, Perlis, Malaysia; e International Institute of Aquaculture and Aquatic Sciences (I-AQUAS), Universiti Putra Malaysia, Lot 960 Jln Kemang 6, 71050 Port Dickson, Negeri Sembilan, Malaysia; f Department of Crop Science, Faculty of Agricultural and Forestry Sciences, Universiti Putra Malaysia Kampus Bintulu Sarawak, Jalan Nyabau, 97008 Bintulu, Sarawak, Malaysia; g Bioactivity Programme, Natural Products Division, Forest Research Institute Malaysia (FRIM), 52109 Kepong, Selangor Darul Ehsan, Malaysia

**Keywords:** Gallic acid, Antioxidant, Anti-inflammatory, *Labisia pumila*

## Abstract

The article presents data on the quantification of gallic acid (GA) and the assessment of the antioxidant and anti-inflammatory properties of Kacip Fatimah (*Labisia pumila* Benth. & Hook. f.) leaves using various solvents. GA was quantified using high-performance liquid chromatography analysis. Total phenolic content (TPC) was assessed using the Folin-Ciocalteu method. The antioxidant activities were evaluated using xanthine oxidase superoxide (XOD-Superoxide) and 2,2-diphenyl-1-picrylhydrazyl (DPPH) assays, while anti-inflammatory activities were examined through lipoxygenase (LOX) and xanthine oxidase (XOD) inhibition assays. Results showed that the water-extracted sample had the highest GA and TPC among the solvents tested, along with the strongest inhibition activities in the XOD-Superoxide and DPPH assays. Both water and ethanol extracts showed significant inhibitory activities in the LOX assay but were inactive in the XOD assay. These findings suggest that the bioactivity of *L. pumila* leaf extract is associated with GA and TPC. GA and TPC strongly correlated with antioxidant and anti-inflammatory activities, except for the XOD assay. The dataset highlights the potential dietary benefits of *L. pumila* leaves as a natural source of antioxidants and anti-inflammatory properties for pharmaceutical and nutraceutical applications.

Specifications Table

**Table:** 

Subject	Biological Sciences / Biochemistry
Specific subject area	Medicinal plant and its pharmacological properties
Data format	Raw, Analysed
Type of data	Table
Data collection	The extraction process was conducted in a water bath. Gallic acid (GA) quantification was performed using high-performance liquid chromatography (HPLC) analysis. The antioxidant and anti-inflammatory activities were measured using a spectrophotometer, based on specific absorbance values for each assay.
Data source location	The *Labisia pumila* Benth. & Hook. f. dried leaves were supplied by the Forest Research Institute Malaysia (FRIM) throughout the experiment.
Data accessibility	Repository name: Mendeley data Data identification number: 10.17632/k975xr6g9y.3[1] Direct URL to data: https://data.mendeley.com/datasets/k975xr6g9y/3

## 1 Value of the Data

• This dataset provides valuable insights into the antioxidant and anti-inflammatory properties of gallic acid derived from *L. pumila* leaf extract through different solvent extractions.
• The data allows researchers to understand the effectiveness of different solvents in extracting bioactive compounds and their impact on the antioxidant and anti-inflammatory activities of the *L. pumila* leaf extract.
• This information can be utilised to further explore the therapeutic applications of *L. pumila* leaves, develop novel formulations, and design targeted studies to investigate its potential benefits in preventing or managing oxidative stress-related disorders and inflammation.

## 2 Data Description

The dataset associated with this study has been deposited on Mendeley and can be accessed using the following link: https://data.mendeley.com/datasets/k975xr6g9y/3

The file is named "Dataset of Gallic Acid, Antioxidant, and Anti-inflammatory Activities of *L. pumila*". It comprises five sheets, each containing distinct sets of data:

• **Sheet 1: Gallic Acid**: This sheet presents data on the GA content of *L. pumila* extracts.
• **Sheet 2: Total Phenolic Content**: This sheet presents data on the total phenolic content (TPC) of *L. pumila* extracts.
• **Sheet 3: Antioxidant**: This sheet presents data on the antioxidant activities of *L. pumila* extracts.
• **Sheet 4: Anti-inflammatory**: This sheet presents data on the anti-inflammatory activities of *L. pumila* extracts.
• **Sheet 5: Statistical Analysis**: This sheet includes the results of the statistical analyses performed on the aforementioned data.

In Table 1, GA was exclusively detected in water and ethanol extracts, with water at 120.00 ± 4.04 mg/g and ethanol at 90.00 ± 5.13 mg/g. TPC varied among solvent extracts: water (211.40 ± 0.41 mg GAE/100 g), ethanol (87.70 ± 0.59 mg GAE/100 g), ethyl acetate (37.10 ± 0.38 mg GAE/100 g), and hexane (32.30 ± 1.22 mg GAE/100 g). Antioxidant activity order for xanthine oxidase superoxide (XOD-Superoxide) and 2,2-diphenyl-1-picrylhydrazyl (DPPH) assays: hexane (68.14 ± 0.89 %, 23.71 ± 1.19 %) < ethyl acetate (81.21 ± 0.56 %, 33.74 ± 0.85 %) < ethanol (91.70 ± 0.82 %, 78.61 ± 1.12 %) < water (97.39 ± 0.33 %, 96.78 ± 0.25 %).

**Table 1:** GA, TPC, antioxidant, and anti-inflammatory activities of *L. pumila* obtained from different solvent extractions.

Solvent Extractions	GA^1^	TPC^2^	XOD-Superoxide^3^	DPPH^3^	XOD^3^	LOX^3^
Water	120.00 ± 4.04 ^a^	211.40 ± 0.41 ^a^	97.39 ± 0.33 ^a^	96.78 ± 0.25 ^b^	2.04 ± 0.10 ^e^	71.48 ± 0.13 ^c^
Ethanol	90.00 ± 5.13 ^b^	87.70 ± 0.59 ^b^	91.70 ± 0.82 ^b^	78.61 ± 1.12 ^c^	8.37 ± 0.11 ^b^	76.13 ± 1.14 ^b^
Ethyl acetate	0.00 ± 0.00 ^c^	37.10 ± 0.38 ^c^	81.21 ± 0.56 ^c^	33.74 ± 0.85 ^d^	3.61 ± 0.07 ^c^	34.09 ± 0.92 ^d^
Hexane	0.00 ± 0.00 ^c^	32.30 ± 1.22 ^d^	68.14 ± 0.89 ^d^	23.71 ± 1.19 ^e^	2.96 ± 0.08 ^d^	29.52 ± 0.78 ^e^
Superoxide dismutase			98.98 ± 0.29 ^a^			
Ascorbic acid				99.41 ± 0.27 ^a^		
Allopurinol					98.27 ± 0.26 ^a^	
NDGA 4						99.58 ± 0.19 ^a^

The values represent the means ± standard deviations of three replicates. Different letters (within a column) indicate significant differences (one-way ANOVA, Tukey's HSD test, *p* < 0.05).

1 mg/g.

2 mg gallic acid equivalent per 100 grams (mg GAE/100 g).

3 percentage ( %).

4 Nordihydroguaiaretic acid.

The anti-inflammatory inhibition results for different solvent extracts are presented in Table 1. The xanthine oxidase (XOD) assay produced the following outcomes: water (2.04 ± 0.10 %), hexane (2.96 ± 0.08 %), ethyl acetate (3.61 ± 0.07 %), and ethanol (8.37 ± 0.11 %). In the lipoxygenase (LOX) assay, both water (71.48 ± 0.13 %) and ethanol (76.13 ± 1.14 %) extracts exhibited higher inhibitory activity. Conversely, ethyl acetate (34.09 ± 0.92 %) and hexane (29.52 ± 0.78 %) extracts demonstrated lowest inhibition activity.

The correlation results were summarised in Table 2. GA and TPC are highly correlated (*r* = 0.899). Strong correlations were observed between GA and XOD-Superoxide (*r* = 0.908), GA and DPPH (*r* = 0.992), GA and LOX (*r* = 0.958), as well as between TPC and XOD-Superoxide (*r* = 0.828), TPC and DPPH (*r* = 0.902), and TPC and LOX (*r* = 0.745). In contrast, both GA (*r* = 0.203) and TPC (*r* = –0.240) exhibited weak correlation with XOD.

**Table 2:** The Pearson correlation coefficients (r) between GA, TPC, and the antioxidant and anti-inflammatory activities of *L. pumila* leaves obtained from different solvent extractions.

Pearson correlation (r)	GA	TPC	XOD-Superoxide	DPPH	XOD	LOX
GA	1	0.899 *	0.908 *	0.992 *	0.203	0.958 *
TPC	0.899 *	1	0.828 *	0.902 *	–0.240	0.745 *

⁎ Correlation is significant at *p* < 0.01 (2-tailed).

## 3 Experimental Design, Materials and Methods

### 3.1 Collection of plant material and preparation

The Natural Product Division, FRIM, consistently supplied dried *L. pumila* leaves for the experiment. The leaves stock was dried in a 50°C oven for 24 h.

### 3.2 Extraction process

The extraction process employed a water bath and involved four solvents: water, ethanol, ethyl acetate, and hexane. The process maintained consistent parameters, including a solid-to-solvent concentration ratio of 10 mg/mL, a duration of 4 h, and a temperature of 40°C. For extraction, 10 g of dried *L. pumila* leaves were placed in a 500 mL round-bottom flask. Filtration of the extract was performed using a Buchner filter under vacuum. The water extract was stored at –80°C before freeze-drying, while the organic solvent extracts were kept at room temperature for solvent recovery. The water extract underwent freezing to remove the solvent, while the ethanol, ethyl acetate, and hexane extracts were recovered using a rotary evaporator under vacuum. The evaporation process was conducted at 40°C to minimise the potential degradation of phytochemicals in the samples.

### 3.3 Quantification of gallic acid compound

For the water-extracted sample, 1 mg of the dried extract was dissolved in 1 mL of water with 50 % methanol. In contrast, the dried extracts of ethanol, ethyl acetate, and hexane (1 mg each) were dissolved in 1 mL of methanol. HPLC analysis was performed, which included a quaternary pump, an autosampler, and a photodiode array detector. Compound detection and chromatographic profiles utilised a reversed-phase C_18_ column at room temperature with scanning in the range of 190 to 400 nm and flow rate of 1 mL/min. A gradient mobile phase consisting of 0.1 % orthophosphoric acid and 100 % acetonitrile was employed.

### 3.4 Total phenolic content

To measure TPC as previously described [2], 50 µL of the supernatant extract was combined with 100 µL of Folin-Ciocalteu reagent (0.1 mL/0.9 mL) in 96-well microtiter plates. The plate was kept at room temperature for 5 min. Subsequently, 100 µL of sodium bicarbonate solution (60 mg/mL) was added to the mixture, followed by further incubation at room temperature for 90 min. The absorbance of the extracts was measured at 725 nm using a spectrophotometer.

### 3.5 Antioxidant analysis

#### 3.5.1 XOD-superoxide radical scavenging activity

The XOD-Superoxide radical scavenging activity was conducted following the protocol described by Phuwapraisirisan et al. [3]. To prepare the stock solutions, the extracts were dissolved in ethanol to achieve a concentration of 50 mg/mL. The reaction mixture was created by dissolving 0.53 g of sodium carbonate (pH 10.2), 4 mg of EDTA, and 2 mg of xanthine in a 0.025 mM 4-nitro blue tetrazolium chloride (NBT) solution (100 mL of 4.1 mM/L). The NBT solution was prepared by combining 3.15 g of Tris hydrochloride, 0.1 g of magnesium chloride, 15 mg of 5-bromo-4-chloro-3-indolyl phosphate, and 34 mg of NBT in 100 mL of distilled water. The mixture was refrigerated at 4°C. For the assay, 5 µL of the stock solution was mixed with 995 µL of the reaction mixture in a microcuvette. Next, 0.1 µL of XOD (1 × 10^–3^ U/mL) was added to initiate the reaction. The mixture was measured at an absorbance of 560 nm for 2 min. Superoxide dismutase enzyme served as a positive control, while the reaction mixture (5 µL) acted as the negative control.

#### 3.5.2 DPPH radical scavenging activity

The method outlined by Brand-Williams et al. [4] was employed to perform the DPPH radical scavenging activity. A stock solution of the extracts was prepared in methanol at a concentration of 0.5 mg/mL. The reaction mixture, composed of 4 mL of the solution and 1 mL of DPPH, was stored in a 5 mL screw-cap vial. After shaking, the mixture was left at room temperature for 3 min. The absorbance of the mixture was measured at 520 nm using a spectrophotometer. The absorbance of the negative control (methanol) and positive control (ascorbic acid) were obtained by replacing the solution.

### 3.6 Anti-inflammatory analysis

#### 3.6.1 XOD inhibition assay

The XOD inhibition assay was conducted based on the procedure outlined in Owen and Johns [5]. The extracts were dissolved in dimethyl sulfoxide (DMSO) to create stock solutions at a concentration of 20 mg/mL. A mixture comprising 10 µL of the working solution of the crude extract, 10 µL of XOD solution, and 130 µL of potassium phosphate buffer at pH 7.5 was incubated at 25°C for 15 min. The reaction was initiated by adding 100 µL of xanthine solution as the substrate. The enzymatic conversion of xanthine to uric acid and hydrogen peroxide was measured at an absorbance of 295 nm using a spectrophotometer. In the control mixture, the solution was substituted with an equal volume of 0.5 % DMSO. Allopurinol served as the positive control to validate the assay's performance.

#### 3.6.2 LOX inhibition assay

The LOX inhibition assay was conducted following the procedure outlined by Azhar-Ul-Haq et al. [6]. The extracts were dissolved in DMSO to create stock solutions with a concentration of 20 mg/mL. A mixture was prepared consisting of 10 µL of the working solution of the crude extract, 20 µL of soybean LOX solution, and 160 µL of sodium phosphate buffer at pH 8.0. The mixture was incubated at 25°C for 15 min. To initiate the reaction, a substrate solution of sodium linoleic acid (10 µL) was added. The enzymatic conversion of sodium linoleic acid to (9Z,11E)-13-hydroperoxyoctadeca-9,11-dienoate was monitored by measuring the changes in absorbance at 234 nm using a spectrophotometer over a six-minute period. In the control mixture, the solution was replaced with an equal volume of 0.5 % DMSO. The positive control used in this assay was NDGA.

### 3.7 Statistical analysis

All assays were performed in triplicate, and the mean and standard deviation were calculated for each assay. To evaluate statistical significance, a one-way ANOVA with Tukey's HSD test was conducted to compare the different solvents. The statistical analyses were carried out using the SPSS tool, and a significance level of *p <* 0.05 was used to determine the significance of the results. For correlation analysis, the Pearson correlation coefficient (r) was employed, and a significance level of *p <* 0.01 was applied.

## 4 Limitations

Not applicable.

## Ethics Statement

The authors have read and follow the ethical requirements for publication in Data in Brief and confirming that the current work does not involve human subjects, animal experiments, or any data collected from social media platforms.

## CRediT authorship contribution statement

**Mohd Azrie Awang:** Conceptualization, Methodology, Writing – original draft, Software, Supervision, Writing – review & editing. **Mohammad Amil Zulhilmi Benjamin:** Writing – original draft, Software, Writing – review & editing. **Adilah Anuar:** Validation. **Mohd Fakhrulddin Ismail:** Visualization. **Shiamala Devi Ramaiya:** Conceptualization, Validation. **Siti Nur Aisyah Mohd Hashim:** Data curation, Investigation.

## Data Availability

Dataset of gallic acid quantification and their antioxidant and anti-inflammatory activities of different solvent extractions from Kacip Fatimah (Labisia pumila Benth. & Hook. f.) leaves (Original data) (Mendeley Data).
